# Non-Small-Cell Lung Cancer Patients with a High Predicted Risk of Irradical Resection: Can Chemoradiotherapy Offer Similar Survival?

**DOI:** 10.1245/s10434-021-10982-3

**Published:** 2021-10-30

**Authors:** W. Hugo van Joolingen, Marnix J. A. Rasing, Max Peters, Anne S. R. van Lindert, Linda M. de Heer, Mieke J. Aarts, Joost J. C. Verhoeff, Peter S. N. van Rossum

**Affiliations:** 1 Department of Radiation Oncology, University Medical Center Utrecht, Utrecht, The Netherlands; 2 Department of Pulmonology, University Medical Center Utrecht, Utrecht, The Netherlands; 3 Department of Cardiothoracic Surgery, University Medical Center Utrecht, Utrecht, The Netherlands; 4 Netherlands Cancer Registry, Netherlands Comprehensive Cancer Organization, Utrecht, The Netherlands

## Abstract

**Purpose:**

Irradical resection of non-small-cell lung cancer (NSCLC) is a detrimental prognostic factor. Recently, Rasing et al. presented an internationally validated risk score for pre-treatment prediction of irradical resection. We hypothesized that chemoradiation therapy (CRT) could serve as an alternative approach in patients with a high risk score and compared overall survival (OS) outcomes between surgery and CRT.

**Methods:**

Patients from a population-based cohort with stage IIB–III NSCLC between 2015 and 2018 in The Netherlands were selected. Patients with a ‘Rasing score’ > 4 who underwent surgery were matched with patients who underwent CRT using 1:1 nearest-neighbor propensity score matching. The primary endpoint of OS was compared using a Kaplan–Meier analysis.

**Results:**

In total, 2582 CRT and 638 surgery patients were eligible. After matching, 523 well-balanced pairs remained. Median OS in the CRT group was 27.5 months, compared with 45.6 months in the surgery group (HR 1.44, 95% CI 1.23–1.70, *p* < 0.001). The 114 surgical patients who underwent an R1–2 resection (21.8%) had a worse median OS than the CRT group (20.2 versus 27.5 months, HR 0.77, 95% CI 0.61–0.99, *p* = 0.039).

**Conclusion:**

In NSCLC patients at high predicted risk of irradical resection, CRT appears to yield inferior survival compared with surgery. Therefore, choosing CRT instead of surgery cannot solely be based on the Rasing score. Since patients receiving an R1–2 resection do have detrimental outcomes compared with primary CRT, the treatment decision should be based on additional information, such as imaging features, comorbidities, patient preference, and the surgeon’s confidence in achieving an R0 resection.

**Supplementary Information:**

The online version contains supplementary material available at 10.1245/s10434-021-10982-3.

Lung cancer is the leading cause of cancer-related deaths in both men and women internationally.^1^ Non-small-cell lung cancer (NSCLC) is the most common histology for lung tumors, accounting for 80–85% of cases.^2^ Resection remains the cornerstone of treatment of early and locally advanced NSCLC, and surgery is currently recommended in all resectable tumors.^3^ For those treated with surgery, the aim is to perform a radical (R0) resection, as irradical (R1–2) resection is associated with a considerably worse prognosis. Irradical resection has been associated with hazard ratios (HRs) for death between 1.5 and 8.2 compared with radical resection.^4,5^ Chemoradiation therapy (CRT) is the accepted alternative approach with curative intent in patients with locally advanced NSCLC who are deemed unresectable.^6,7^

Recently, a prediction score for irradical resection has been developed in the Netherlands and validated in the United States of America, by Rasing et al.^8^ This model was developed using data from the Netherlands Cancer Registry, which resulted in a multivariable logistic regression model for the prediction of irradical resection with a good discriminative performance (external c-statistic 0.71). Predictive parameters included histology, clinical T-stage, clinical N-stage, planned extent of resection (e.g., lobectomy, pneumonectomy), and surgical approach (thoracoscopic, open). Patients with a Rasing score > 4 were deemed at high risk, as their individual predicted and observed probability of an R1–2 resection was > 13% in all these patients, and 19% on average.^8^

We hypothesized that CRT could serve as an alternative approach in patients with a high risk of an irradical resection as predicted by the Rasing score.^8^ Therefore, the primary aim of this study was to compare overall survival (OS) between surgery and CRT in those patients. A secondary aim was to identify potentially varying OS outcomes for CRT versus surgery across different patient subgroups.

## Materials and Methods

This study was a population-based retrospective cohort study. Institutional review board approval was obtained and the need for written informed consent was waived, as all patient information in the database used was anonymized.

### Study Population

#### Surgery Group

The surgery group used in this study was formed using a group of patients described in Rasing et al.^8^ These patients were identified using the national database of the Netherlands Cancer Registry (managed by the Netherlands Comprehensive Cancer Organization). All newly diagnosed cancer cases in the Netherlands are registered in this database by independent data managers. The database contains in-depth patient, tumor, and treatment characteristics, which are gathered based on information from electronic medical records, the Pathological Anatomical National Automated Archive and the national registry of hospital discharge diagnoses and diagnosis-treatment combinations (DBC). All patients diagnosed with NSCLC between January 1, 2015, and December 31, 2018 and treated surgically were included for analysis.

The predicted probability (*p*) of an R1–2 resection was calculated using the following formula:^8^

$$\begin{gathered} ln \, \left( {p \, / \, \left( {1 \, {-} \, p} \right)} \right) \, = \, - 2.54 \, + \, ln\left( {0.69} \right) \, * \, \left[ {adenocarcinoma} \right] \, + \, ln\left( {0.79} \right) \, * \, \left[ {other \, histology} \right] \, + \, ln\left( {1.98} \right) \, * \, \left[ {cT2} \right] \, + \hfill \\ ln\left( {2.90} \right) \, * \, \left[ {cT3} \right] \, + \, ln\left( {4.50} \right) \, * \, \left[ {cT4} \right] \, + \, ln\left( {0.45} \right) \, * \, \left[ {lobar \, or \, bilobar} \right] \, + \, ln\left( {0.86} \right) \, * \, \left[ {sleeve \, lobectomy} \right] \, + \, ln\left( {0.69} \right) \hfill \\ * \, \left[ {pneumonectomy} \right] \, + \, ln\left( {1.42} \right) \, * \, \left[ {open \, approach} \right] \, + \, ln\left( {1.96} \right) \, * \, \left[ {cN1} \right] \, + \, ln\left( {1.73} \right) \, * \, \left[ {cN2 \, or \, cN3} \right] \hfill \\ \end{gathered}$$

where ‘yes = 1’ and ‘no = 0’. According to the published nomogram, patients with a predicted probability of > 13% (or Rasing score > 4) were considered at high risk for an R1–2 resection.

Exclusion criteria used in the study by Rasing et al. included age < 18 years, cM1 stage, cTis, cT0, neoadjuvant chemotherapy or preoperative radiotherapy, or both, carcinoid histology and time interval from diagnosis to resection exceeding 180 days.^8^ Patients with a Rasing score higher than 4 were considered at high risk, in concordance with Rasing et al., and selected for this study. High-risk patients with a clinical stage of I or IIA were excluded. Additionally, patients with a time interval from diagnosis to the beginning of treatment of 0 days, indicating no biopsy prior to surgery, or > 90 days, were excluded.

#### Chemoradiation Therapy Group

The same data set and time period were used to identify patients who underwent CRT. Patients were considered to have received CRT if they received both chemo- and radiotherapy, with chemotherapy being administered first or within 10 days of the first radiotherapy fraction, and with < 120 days between the first dosage of chemotherapy and radiotherapy. Exclusion criteria were age < 18 years, cM1 stage, cN3 stage, cTis, cT0, overall clinical stage I or IIA, carcinoid histology, a time interval from diagnosis to the beginning of treatment of 0 days or > 90 days, and a duration of radiotherapy of < 26 or > 50 days.

### Study Parameters

Variables analyzed for both groups include age, sex, WHO performance status, previous history of malignancy, localization and lateralization of the tumor, tumor histology, cT and cN stages, and overall staging. For the surgery group, completeness of resection (R0 or R1–2), extent of surgery, surgical approach and whether adjuvant chemotherapy was administered were additional variables. For surgical approach, the intended approach was recorded (with conversion to open surgery counting as scopic approach). Type of chemoradiation therapy (sequential or concurrent) was an additional variable in the chemoradiation group. Outcome variables were vital status at the end of follow-up and time duration in days of follow-up from diagnosis. As the database used does not register the ethnicity or race of patients, no data regarding the distribution of ethnicity or race of patients was available for analysis.

### Statistical Analysis

Continuous variables are presented with mean ± standard deviation (SD). Differences in baseline between the two groups in continuous variables were assessed with an unpaired *T*-test or Mann–Whitney *U* test, depending on the normality of the data distribution. Differences in nominal and ordinal categorical variables were assessed with a *χ*^2^ test and the Mann–Whitney *U* test, respectively.

Missing data in the chemoradiation group were assumed to be missing at random, and handled using multiple imputation by chained equations, creating 20 new data sets. The most representative imputation set was used, determined via survival modeling of all imputation sets separately and comparing them with the pooled OS model for all 20 imputed data sets.

The imputed data set of CRT patients was matched to the group of high-risk patients who had undergone surgery identified in the Rasing study using propensity score matching. A logistic regression model was performed to determine a propensity score for each patient in which the variables age, sex, WHO performance score, history of malignancy, lateralization and localization of the tumor, histology, and cT- and cN-staging were accounted for. Patients from both groups were matched (1:1) according to nearest-neighbor matching without replacement. Within-pair difference was minimized by setting a caliper of 0.1 of the standard deviation of the logit of the propensity score. Standardized mean differences (SMD) were calculated to check the balance of the match, with a threshold of 0.1 considered well balanced. Additionally, the groups were compared on all variables accounted for in the matching process, similarly to the baseline comparison, to further check the balance of the match. Imputation of missing data and propensity score matching were performed with “mice” and “MatchIt” packages in R 4.0.3 software (The R Foundation for Statistical Computing, Vienna, Austria).

To compare the matched groups, OS analysis was performed with Kaplan–Meier survival analysis. In addition to the main survival analysis, subgroup analyses were performed using Cox regression models to identify potential differences in hazard ratios across subgroups. To compare OS for CRT with those receiving irradical resection, Kaplan–Meier survival analysis was performed. Finally, to assess whether results would be different when a different Rasing score threshold for high risk was chosen, a sensitivity analysis was performed, using a risk score of > 5 as cut-off for patient selection. Analysis was performed using SPSS version 25.0 (IBM Corp, IBM SPSS Statistics for Windows, Armonk, NY). A *p* value < 0.05 was considered statistically significant.

## Results

**Fig. 1:**
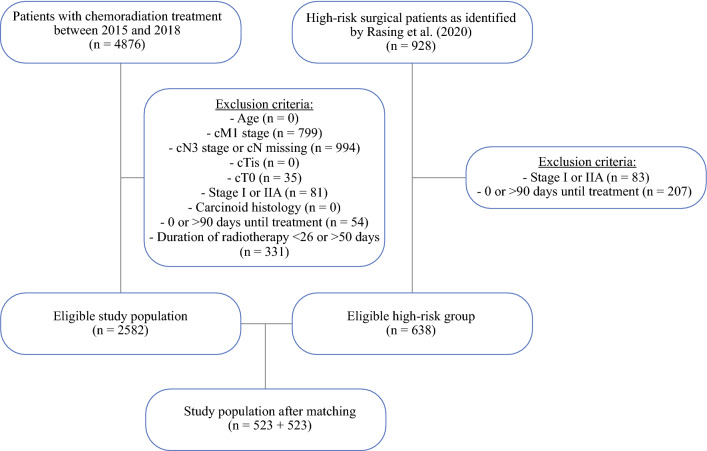
Flowchart of study profile and patient selection

Among 7156 surgically treated patients for stage I–III NSCLC between 2015 and 2018, 928 (13.0%) had a high predicted risk (Rasing score > 4). Of these, 182 (19.6%) underwent an irradical resection. For this study, 290 patients were excluded based on the exclusion criteria. Additionally, a total of 4876 patients treated with CRT for stage I–III NSCLC between 2015 and 2018 were identified. Of these, 2294 were excluded based on the exclusion criteria (Fig. 1).

Before propensity score matching, major statistical differences across most baseline variables were observed between both groups (Table 1). After propensity score matching, 523 pairs of patients remained, and were included for further analysis. SMDs were below the 0.1 threshold, indicating a well-balanced match. On average, patients were 67.5 years old, the majority were male and most had a WHO performance status of 0–1. The majority (60.4%) of patients had a cT4 tumor, and 30.4% and 17.1% were staged cN1 and cN2, respectively. Two thirds of patients had an overall clinical stage IIIA, with 21.4% and 12.5% having stage IIB and IIIB disease, respectively. Of the surgical patients, 114 (21.8%) underwent an R1–2 resection, whereas an R0 resection was achieved in 409 (78.2%). Most surgical patients (49.3%) underwent a lobectomy or bilobectomy, whereas pneumonectomy, sleeve lobectomy, and sublobar resections were performed in 37.3%, 10.7%, and 2.7%, respectively. The majority (71.5%) of surgical patients underwent an open procedure. Adjuvant chemotherapy was administered in 206 (39.4%) of surgical patients. Of the CRT patients, 144 (27.5%) and 379 (72.5%) received sequential and concurrent CRT, respectively.

**Table 1:** Baseline table (before and after matching)

Characteristic	Unmatched groups			After matching		
	Surgery group (*n* = 638)	CRT group (n=2582)	*p* value	Surgery group (*n* = 532)	CRT group (*n* = 532)	*p* value
*Male sex*	443 (69.4)	1494 (57.9)	<0.001	343 (65.6)	333 (63.7)	0.518
*Age (years)*	67.6 ± 8.3	66.1 ± 8.9	<0.001	67.6 ± 8.5	67.5 ± 9.1	0.849
*WHO performance status*			0.015			0.121
WHO 0-1	606 (95.0)	2379 (92.1)		493 (94.3)	480 (91.8)	
WHO 2	25 (3.9)	181 (7.0)		24 (4.6)	38 (7.3)	
WHO 3	7 (1.1)	22 (0.9)		6 (1.1)	5 (1.0)	
*History of malignancy*	122 (19.1)	505 (19.6)	0.803	101 (19.3)	103 (19.7)	0.876
*Tumor location*			<0.001			0.454
Inferior lobe	237 (37.1)	599 (23.2)		158 (30.2)	147 (28.1)	
Other	401 (62.9)	1983 (76.8)		365 (69.8)	376 (71.9)	
*Lateralization*			<0.001			0.495
Left	327 (51.3)	945 (36.6)		250 (47.8)	239 (45.7)	
Right	311 (48.7)	1634 (63.3)		273 (52.2)	284 (54.3)	
Medial	0 (0.0)	1 (0.0)		0 (0.0)	0 (0.0)	
Both sides	0 (0.0)	2 (0.1)		0 (0.0)	0 (0.0)	
*Tumor histology*			<0.001			0.570
Squamous cell carcinoma	403 (63.2)	1085 (42.0)		303 (57.9)	293 (56.0)	
Adenocarcinoma	177 (27.7)	1053 (40.8)		162 (31.0)	161 (30.8)	
Other types	58 (9.1)	444 (17.2)		58 (11.1)	69 (13.2)	
*Clinical T-stage*			<0.001			0.109
cT1	3 (0.5)	441 (17.1)		3 (0.6)	1 (0.2)	
cT2	111 (17.4)	523 (20.3)		83 (15.9)	71 (13.6)	
cT3	207 (32.4)	455 (17.6)		133 (25.4)	123 (23.5)	
cT4	317 (49.7)	1163 (45.0)		304 (58.1)	328 (62.7)	
*Clinical N-stage*			<0.001			0.807
cN0	294 (46.1)	443 (17.2)		270 (51.6)	279 (53.3)	
cN1	258 (40.4)	233 (9.0)		167 (31.9)	151 (28.9)	
cN2	86 (13.5)	1906 (73.8)		86 (16.4)	93 (17.8)	
*Clinical TNM-stage*			<0.001			0.978
IIB	154 (24.1)	161 (6.2)		110 (21.0)	114 (21.8)	
IIIA	421 (66.0)	1391 (53.9)		350 (66.9)	341 (65.2)	
IIIB	63 (9.9)	1030 (39.9)		63 (12.0)	68 (13.0)	

**Fig. 2:**
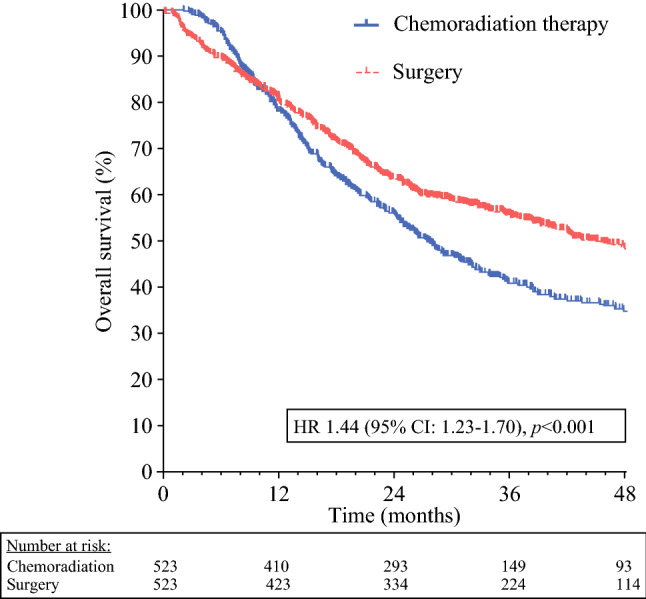
Kaplan–Meier curve comparing OS after chemoradiation therapy versus surgery in NSCLC patients with a high predicted risk of an irradical (R1–2) resection

**Fig. 3:**
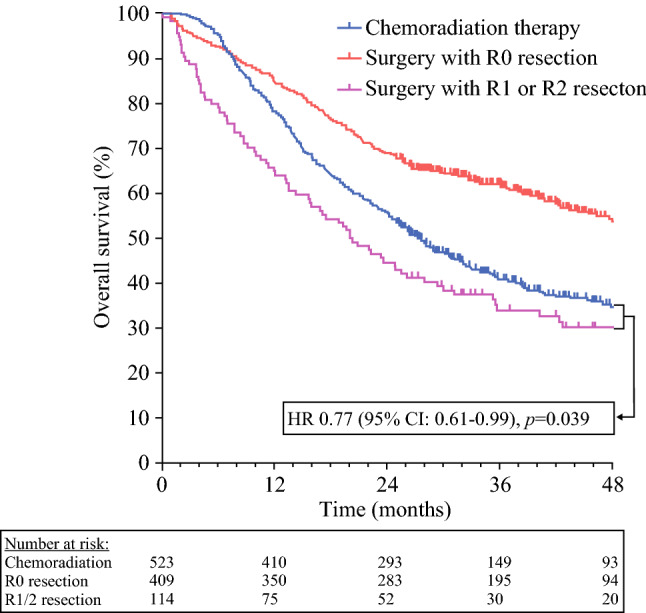
Kaplan–Meier curve comparing OS after chemoradiation therapy versus surgery with R0 resection and surgery with R1 or R2 resection in NSCLC patients with a high predicted risk of an irradical (R1–2) resection

After a median follow-up of 28.2 months, the median OS among patients treated with surgery was 45.6 months, compared with 27.5 months in the CRT group (log-rank *p* < 0.001; Fig. 2). Cox regression analysis revealed an HR of 1.44 (95% CI 1.23–1.70). The CRT group had better OS than those in the surgery group who ended up receiving an R1 or R2 resection (*n* = 114), with an estimated median OS of the latter group of 20.2 months (*p* = 0.039; Fig. 3). The HR between the CRT group and the patients who received an R1 or R2 resection was 0.77 (95% CI 0.61–0.99).

**Fig. 4:**
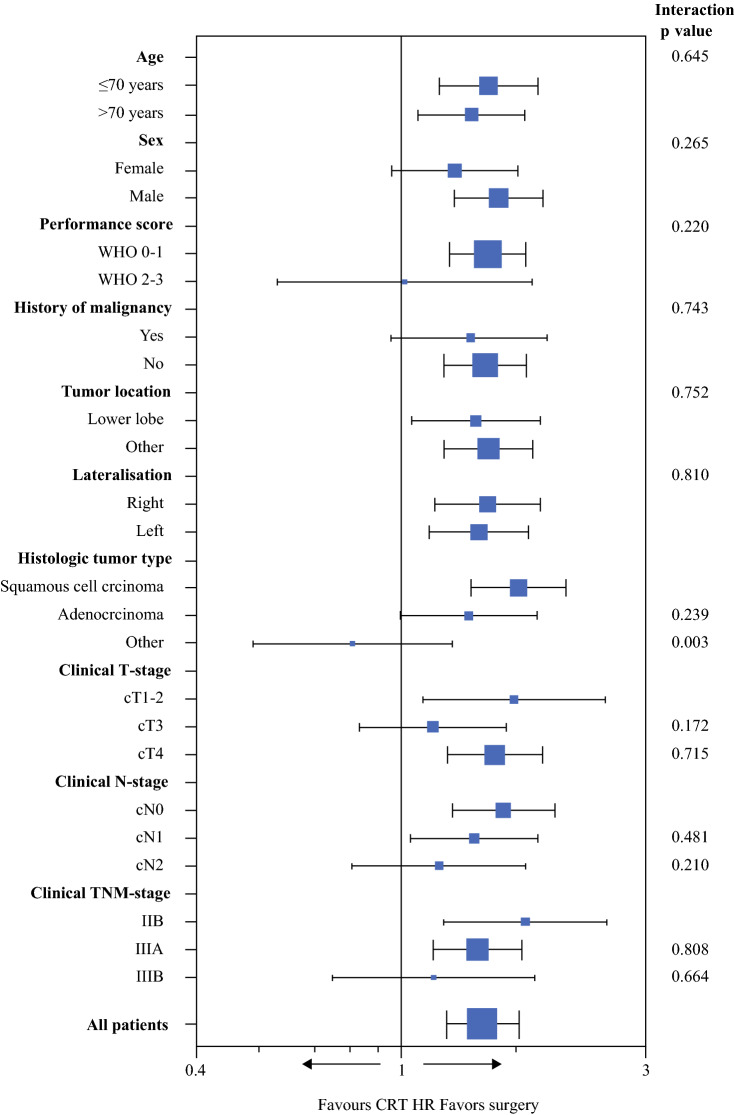
Forest plot comparing hazard ratios of survival after chemoradiation therapy versus surgery in NSCLC patients with a high predicted risk of an irradical (R1–2) resection, organized per subgroup and compared using Cox regression

The HRs for OS across subgroups are presented in Fig. 4. A subgroup of patients with a histology other than adenocarcinoma or squamous cell carcinoma had a significantly different (interaction *p* = 0.003) HR for CRT versus surgery, with no significant superiority of surgery nor CRT in that subgroup. Other subgroups, including those based on age, WHO performance status, and clinical staging, did not modify the overall observed effect of CRT versus surgery on OS. Sensitivity analysis using a Rasing score cut-off for high risk of > 5 (instead of > 4) yielded similar outcomes regarding the HR of CRT versus surgery on OS (HR 1.36, 95% CI 1.09–1.71; Supplemental Table 1 and Supplemental Fig. 1).

## Discussion

To improve patient selection for NSCLC surgery, the Rasing score was developed in 7156 patients and validated in 82,235 patients for individualized pre-treatment prediction of an irradical (R1–2) resection.^8^ In the patient group with a high predicted risk of such irradical resection (score > 4), the current study demonstrates that CRT as an alternative treatment approach resulted in diminished OS compared with surgery. Specifically, the median survival for surgical treatment was found to be 45.6 months, compared with 27.5 months for CRT. However, the subgroup who indeed underwent R1 or R2 resection (21.8%) were found to have a significantly worse survival compared with CRT. These findings support the current standard of surgery as the preferred treatment, even in high-risk cases, provided that an R0 resection is achieved. CRT remains a good alternative for patients in whom an R1 or R2 resection is expected. The Rasing score^8^ may aid in estimating this risk, but additional clinical information and judgement (based on parameters not measured in the Rasing score), such as imaging features, comorbidities, assessment by the surgeon, and patient preference remain important for final treatment decision-making.

The current treatment for NSCLC mainly relies on surgery, especially for stage I and II tumors.^9,10^ Five-year OS rates for surgery in early stages of the disease have been reported to be 73–78%.^11^ For stage III NSCLC a median survival of about 2 years has been achieved.^12,13^ The surgical group in the current study had a favorable median OS of 45.6 months (3.8 years). A likely explanation for this superior survival outcome is stricter selection of patients compared with other studies; for example, patients with earlier stage disease, better overall patient condition, and more patients who received adjuvant therapy. Unfortunately, irradical resection remains a considerable impediment, resulting in significantly worse survival, as found in many reports, including the current study.^4,5,14–19^

When tumors are deemed irresectable due to local advancement or a patient has contra-indications for surgical treatment, radiation-based therapy can be used as an alternative.^9,10,20^ Over the years, this treatment approach has improved from simple thoracic radiotherapy to advanced image-guided radiotherapy and platinum-based chemotherapy to improve survival. Radiation-based therapy when used as an alternative for surgery in clinical stage I patients can achieve a local tumor-control rate of more than 85% at 5 years.^10^ For locally advanced NSCLC (stage IIIA–B), multiple trials reported median OS around 2 years and 5-year OS rates of 15–20%.^6,10,20^ These results are comparable with the median OS of 27.5 months (2.3 years) for patients treated with CRT in the current study.

Preoperatively estimating the chance of an irradical resection may be challenging. In an aim to aid in the clinical decision-making, the Rasing score was developed and validated using large population-based databases.^8^ Predictive parameters for an R1–2 resection included histology, clinical T-stage, clinical N-stage, planned extent of resection (e.g., lobectomy, pneumonectomy), and surgical approach (i.e., thoracoscopic, open). Patients with a Rasing score > 4 were deemed at high risk and were the population of interest in the current study. When using the Rasing score, it is of crucial importance to realize that no causal relationship can be inferred from the prediction model (or any other prediction model). For this reason, the risk score should not be inappropriately used to plan the type of surgery. Rather, the model can be appropriately used after staging has been performed and a surgical plan has been made, to then predict the individual risk of an R1–2 resection. The team of physicians can then use the individually calculated risk of an R1–2 resection to optimize or reconsider the proposed treatment approach.

Comparison of surgery versus CRT was not previously performed for the specific group with a high predicted risk of irradical resection. This study has compared both treatment methods via propensity score matching of patients identified as high-risk patients in the study by Rasing et al. to patients treated with chemoradiation therapy. The overall median survival found in both groups supports the use of surgery in most cases, with a hazard ratio of 1.44 (95% CI 1.23–1.70). The subgroup analysis did not find any subgroups with a clear benefit of CRT. On the other hand, the overall superiority of surgery was not evident in some subgroups of patients where the confidence intervals around the subgroup HRs contained 1.0. This included those with WHO performance scores 2–3 and patients with cN2 or stage IIIB disease.

The results of this study should be interpreted with consideration of their limitations. First, due to the limited data available, this study could only assess overall survival, and not ascertain other outcomes, such as progression-free survival. Other outcomes, such as burden of treatment or quality of life, were not reported either, and therefore information on the comparison of treatments on those grounds is still lacking. Secondly, as this was not a randomized controlled trial, a risk of selection bias remains. The known confounders have been corrected using propensity score matching, but residual unknown confounders could remain. Potential confounders could include tumor characteristics on imaging, surgeon and/or team experience level, hospital volume, and patient comorbidities. The current study also has considerable strengths, including the relatively large sample size, the population-based design resulting in higher external validity, and the possible generalization of the results to the international context due to the international validation of the Rasing score.

In conclusion, CRT for selected patients with stage IIB–III NSCLC at a high predicted risk for irradical resection using the internationally validated Rasing risk score^8^ resulted in worse OS compared with surgery. Consequently, the treatment decision of surgery versus CRT cannot solely be based on that risk score. Since patients who end up receiving an R1 or R2 resection do have inferior outcomes compared with primary CRT, the treatment decision should be based on additional information not covered by the Rasing score, such as imaging features, comorbidities, patient preference, and surgeon’s confidence in achieving an R0 resection.

## Supplementary Information

Below is the link to the electronic supplementary material.

Supplementary file1 (PNG 111 KB)

Supplementary file2 (DOCX 19 KB)
