# Supplementary figures and images for: Non-Small-Cell Lung Cancer Patients with a High Predicted Risk of Irradical Resection: Can Chemoradiotherapy Offer Similar Survival?
Source: Ann Surg Oncol. 2021 Oct 30;29(3):1807–14. doi: 10.1245/s10434-021-10982-3 (PMC8810471; doi:10.1245/s10434-021-10982-3)

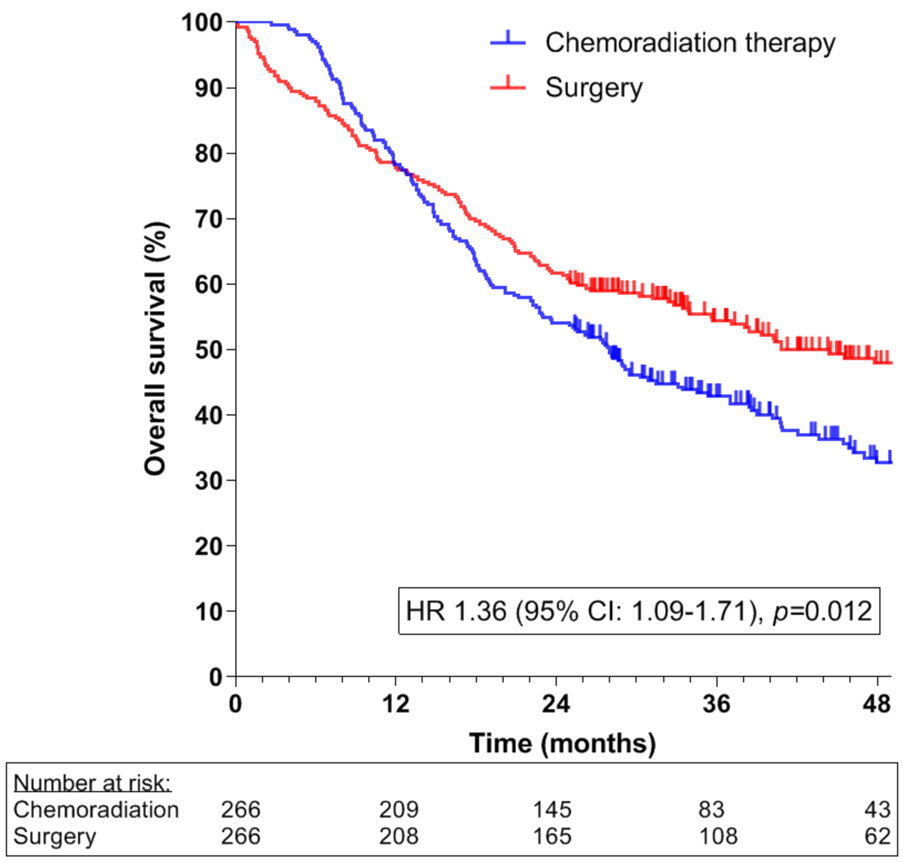

Supplement: Supplementary file 1 — Supplementary file1 (PNG 111 KB) [file 10434_2021_10982_MOESM1_ESM.png]
