# Supplementary material for: Non-Small-Cell Lung Cancer Patients with a High Predicted Risk of Irradical Resection: Can Chemoradiotherapy Offer Similar Survival?
Source: Ann Surg Oncol. 2021 Oct 30;29(3):1807–14. doi: 10.1245/s10434-021-10982-3 (PMC8810471; doi:10.1245/s10434-021-10982-3)
Supplement: Supplementary file 2 — Supplementary file2 (DOCX 19 KB) [file 10434_2021_10982_MOESM2_ESM.docx]

**SUPPLEMENTAL TABLES**

**Supplementary Table 1** Baseline table (before and after matching) for the sensitivity analysis, including NSCLC patients with a high risk of irradical resection defined as Rasing score >5.

|  | ***Unmatched groups*** | | | ***After matching*** | | |  |
| --- | --- | --- | --- | --- | --- | --- | --- |
| ***Characteristic*** | ***Rasing >5 surgery group (n=295)*** | ***CRT group***  ***(n=2582)*** | ***p value*** | ***Rasing >5 surgery group***  ***(n=266)*** | ***CRT group***  ***(n=266)*** | ***p value*** | |
| **Male sex** | 216 (73.2) | 1494 (57.9) | <0.001 | 191 (71.8) | 189 (71.1) | 0.848 | |
| **Age (y)** | 66.8 ± 8.3 | 66.1 ± 8.9 | 0.187 | 66.9 ± 8.4 | 66.7 ± 9.3 | 0.814 | |
| **WHO performance status** |  |  | 0.211 |  |  | 0.158 | |
| WHO 0-1 | 278 (94.2) | 2379 (92.1) |  | 249 (93.6) | 256 (96.2) |  | |
| WHO 2 | 13 (4.4) | 181 (7.0) |  | 13 (4.9) | 10 (3.8) |  | |
| WHO 3 | 4 (1.4) | 22 (0.9) |  | 4 (1.5) | 0 (0.0) |  | |
| **History of malignancy** | 46 (19.0) | 505 (19.6) | 0.813 | 51 (19.2) | 41 (15.4) | 0.252 | |
| **Tumour histology** |  |  | <0.001 |  |  | 0.271 | |
| Squamous cell carcinoma | 201 (68.1) | 1085 (42.0) |  | 173 (65.0) | 180 (67.7) |  | |
| Adenocarcinoma | 65 (22.0) | 1053 (40.8) |  | 64 (24.1) | 50 (18.8) |  | |
| Other types | 29 (9.8) | 444 (17.2) |  | 29 (10.9) | 36 (13.5) |  | |
| **Lateralisation** |  |  | <0.001 |  |  | 0.295 | |
| Left | 181 (61.4) | 945 (36.6) |  | 153 (57.5) | 141 (53.0) |  | |
| Right | 114 (38.6) | 1634 (63.3) |  | 113 (42.5) | 125 (47.0) |  | |
| Medial | 0 (0.0) | 1 (0.0) |  | 0 (0.0) | 0 (0.0) |  | |
| Both sides | 0 (0.0) | 2 (0.1) |  | 0 (0.0) | 0 (0.0) |  | |
| **Tumour location** |  |  | 0.001 |  |  | 0.280 | |
| Inferior lobe | 83 (32.7) | 599 (23.2) |  | 76 (28.6) | 65 (24.4) |  | |
| Other | 171 (67.3) | 1983 (76.8) |  | 190 (71.4) | 201 (75.6) |  | |
| **Clinical T-stage** |  |  | <0.001 |  |  | 0.785 | |
| cT1 | 0 (0.0) | 441 (17.1) |  | 0 (0.0) | 0 (0.0) |  | |
| cT2 | 55 (18.6) | 523 (20.3) |  | 60 (18.8) | 50 (18.8) |  | |
| cT3 | 81 (27.5) | 455 (17.6) |  | 63 (23.7) | 67 (25.2) |  | |
| cT4 | 159 (53.9) | 1163 (45.0) |  | 153 (57.5) | 149 (56.0) |  | |
| **Clinical N-stage** |  |  | <0.001 |  |  | 0.757 | |
| cN0 | 87 (29.5) | 443 (17.2) |  | 87 (32.7) | 89 (33.5) |  | |
| cN1 | 145 (49.2) | 233 (9.0) |  | 116 (46.3) | 106 (39.8) |  | |
| cN2 | 63 (21.4) | 1906 (73.8) |  | 63 (23.7) | 71 (26.7) |  | |
| **Clinical stage** |  |  | <0.001 |  |  | 0.723 | |
| IIB | 53 (18.0) | 161 (6.2) |  | 48 (18.0) | 50 (18.8) |  | |
| IIIA | 190 (64.4) | 1391 (53.9) |  | 166 (62.4) | 167 (62.8) |  | |
| IIIB | 52 (17.6) | 1030 (39.9) |  | 52 (19.5) | 49 (18.4) |  | |

**SUPPLEMENTAL FIGURES LEGENDS**

**Supplementary Figure 1** Kaplan-Meier curve comparing OS after chemoradiation therapy versus surgery in NSCLC patients with a high predicted risk of an irradical (R1-2) resection, defined as Rasing score >5.
